# 3D segmentation of uterine fibroids based on deep supervision and an attention gate

**DOI:** 10.3389/fonc.2025.1522399

**Published:** 2025-03-13

**Authors:** ZhiWei Liu, ChengNv Sun, ChengWei Li, FaJin Lv

**Affiliations:** ^1^ Center of Radiation Oncology, Ganzhou Cancer Hospital, Ganzhou, Jiangxi, China; ^2^ State Key Laboratory of Ultrasound in Medicine and Engineering, College of Biomedical Engineering, Chongqing Medical University, Chongqing, China; ^3^ Department of Radiology, The First Affiliated Hospital of Chongqing Medical University, Chongqing, China

**Keywords:** uterine fibroid, MRI segmentation, deep supervision, attention gate, deep learning

## Abstract

**Introduction:**

The segmentation of uterine fibroids is very important for the treatment of patients. However, uterine fibroids are small and have low contrast with surrounding tissue, making this task very challenging. To solve these problems, this paper proposes a 3D DA- VNet automatic segmentation method based on deep supervision and attention gate.

**Methods:**

This method can accurately segment uterine fibroids in MRI images by convolutional information. We used 3DVnet as the underlying network structure and added a deep monitoring mechanism in the hidden layer. We introduce attention gates during the upsampling process to enhance focus on areas of interest. The network structure is composed of VNet, deep supervision module and attention gate module. The dataset contained 245 cases of uterine fibroids and was divided into a training set, a validation set, and a test set in a ratio of 6:2:2. A total of 147 patients' T2-weighted magnetic resonance (T2WI) images were used for training, 49 for validation, and 49 patients' MR Images were used for algorithm testing.

**Results:**

Experimental results show that the proposed method achieves satisfactory segmentation results. Dice similarity coefficient (DSC), intersection ratio (IOU), sensitivity, precision and Hausdorff distance (HD) were 0.878, 0.784, 0.879, 0.885 and 11.180 mm, respectively.

**Discussion:**

This shows that our proposed method can improve the automatic segmentation accuracy of magnetic resonance image (MRI) data of uterine fibroids to a certain extent

## 1 Introduction

Uterine fibroids are one of the most common benign tumors in women, with an incidence of 20-40% in women during their reproductive period (1). Uterine fibroids can lead to serious morbidity, such as heavy menstrual bleeding and pelvic pressure (2). In addition, they pose a serious threat to women’s health and affect women’s quality of life. Traditional treatment for fibroids is hysterectomy, which can cause physical and emotional pain in women. In recent years, high-intensity focused ultrasound (HIFU) has been widely used and successfully in the treatment of uterine fibroids due to its noninvasive characteristics (3–5). However, whether a fibroids patient is treated with traditional surgery or HIFU, fibroids magnetic resonance image (MRI) segmentation is required before surgery, as MRI is currently considered to be the most accurate imaging technique for detecting and locating fibroids (6). T2-weighted imaging (T2WI) is very important for the diagnosis of gynecological diseases, and examined images can clearly show diseased tissue of the uterus, such as uterine fibroids and evidence of cervical cancer and endometrial cancer. T1-weighted images (T1WIs) enable better observation of anatomical structures. Segmentation of uterine fibroids is usually performed on T2WI MRI images. This needs to be done manually by one or two experienced physicians; manual segmentation of uterine fibroids takes a lot of time, and the segmentation results obtained by doctors vary from person to person, so uterine fibroid MRI segmentation is still a challenging task. The reasons are as follows: (1) The contrast between uterine fibroids and other surrounding tissue is low and the boundary is difficult to distinguish. (2) The area of uterine fibroids on MRI is small, with little available information. (3) The positions of the uterine fibroids are not fixed and it is difficult to segment them.

In recent years, deep learning has become very popular. An increasing number of researchers have begun to use deep learning to classify, recognize and segment medical images, including performing segmentation of uterine fibroids. Regarding deep neural networks, Kurata et al. (7) proposed an improved UNet to segment MRI images of uterine fibroids, replacing the ReLU in each layer in the original UNet with leaky ReLU. A dropout layer was added, batch_size was set to 15, 8 layers of downsampling were used, and finally the Dice similarity coefficient of all uterine fibroids was determined to be 0.820. Tang et al. (8) proposed AR-UNet, which used the ResNet101 deep neural network as the front end of feature extraction to extract semantic information from the image, reducing the number of layers and improving the precision of segmentation. They also introduced an attention gate module between up-sampling and down-sampling and incorporated UNet to build a network structure. Finally, the Dice similarity coefficient of MRI segmentation of uterine fibroids reached 0.904. Zhang et al. (9) proposed HIFUnet, a network with a ResNet101 backbone, global convolutional network (GCN) module, deep multiple atrous convolutions (DMAC) module, sampling, a cascading layer and an output layer. It was used for accurate segmentation of the uterus, uterine fibroids and spine on MRI, and the Dice similarity coefficient of segmentation of uterine fibroids was 0.835. However, these methods have common shortcomings. First, they cannot directly process 3D images, and second, the segmentation accuracy is not high. The existing methods usually convert 3D data into 2D slices through dimensionality reduction or image slicing before training the deep learning model, thus losing considerable image spatial information, which is not conducive to the effective segmentation of 3D images, so the segmentation accuracy is not high. Maike et al (10)proposed a method of automatic segmentation of uterus and uterine fibroids on MRI with 3D nn Unet, and achieved good segmentation results. Therefore, to obtain more in-depth information and better meet the needs of clinical diagnosis, it is necessary to propose a more effective 3D image processing method.

The purpose of this study was to explore the feasibility of automatic 3D VNet segmentation of uterine fibroids based on deep supervision and an attention gate. We applied a 3D deep supervision attention VNet (3D DA-VNet) and optimized some hyperparameters to achieve automatic segmentation. The contributions of this paper are as follows.

1. Compared to existing segmentation methods, we propose a 3D segmentation method to segment uterine fibroids.
2. To solve the problem of disappearance of the gradient and slow convergence, a deep supervision mechanism is introduced.
3. Deep supervision and an attention gate are integrated to improve segmentation accuracy.

The structure of this paper is as follows. Section 2 details the proposed materials and methods, including data preprocessing and model description. Section 3 is mainly about the results and discussion, including parameter settings and evaluation indicators. The conclusion is given in Section 4.

## 2 Materials and methods

### 2.1 Dataset

Data from January 2013 to December 2018 in the first affiliated hospital of the Haifu Minimally Invasive and Non-invasive Treatment Center of the Chongqing Medical University for the ablation treatment of 245 patients with uterine fibroids. The image data parameters are shown in **Table 1**.

**Table 1:** Imaging parameters of MRI T2WI.

parameters	T2WI
TR(ms)	3740
TE(ms)	106.6
FOV(cm)	98.1 × 38
Matrix	512 × 512
Slices	22
Slice thickness(mm)	6
Slice gap(mm)	8
Nex	2

TR, Repetition time; TE, Echo time; FOV, Field of view; Nex, Number of excitations.

### 2.2 Experimental parameters

The experiment was carried out on a TensorFlow 8G NVIDIA GeForce RTX 3070 graphics card, CUDA version 11.3.1. Furthermore, the initial learning rate was set at 2e-4, and the epoch was set at 600. As the epoch increased, the learning rate decreased exponentially at a rate of 0.999. Due to large 3D data and the limitation of device memory, the batch size could only be set to 1; the loss function was Dice (Dice Loss) (11), Adam (12) was used as optimizer, the momentum was 0.9, and PReLU (13) was used as activation function.

### 2.3 Preprocessing

The labels were manually delineated by a physician with 3 years of experience using ITK-SNAP on axial T2WI. The obtained regions of interest were used as the gold standard for segmentation of uterine fibroids. Image preprocessing included normalization, resampling, filling, cropping and random noise. The purpose of normalization was to make the grey values of each image in the training set have the same distribution. Resampling was performed to normalize the voxels of different sizes in the image to the same size. The actual spatial size represented by a single voxel in different images was inconsistent. Because the convolutional neural network only operates in the voxel space, it ignores the size information in the actual physical space. To address this difference, it was necessary to resize different image data in the voxel space to ensure that the actual physical space represented by each voxel was consistent across different image data. The image was then filled and cut to an input size of 128x128x48.

### 2.4 Deep surpervision module

**Figure 1:**
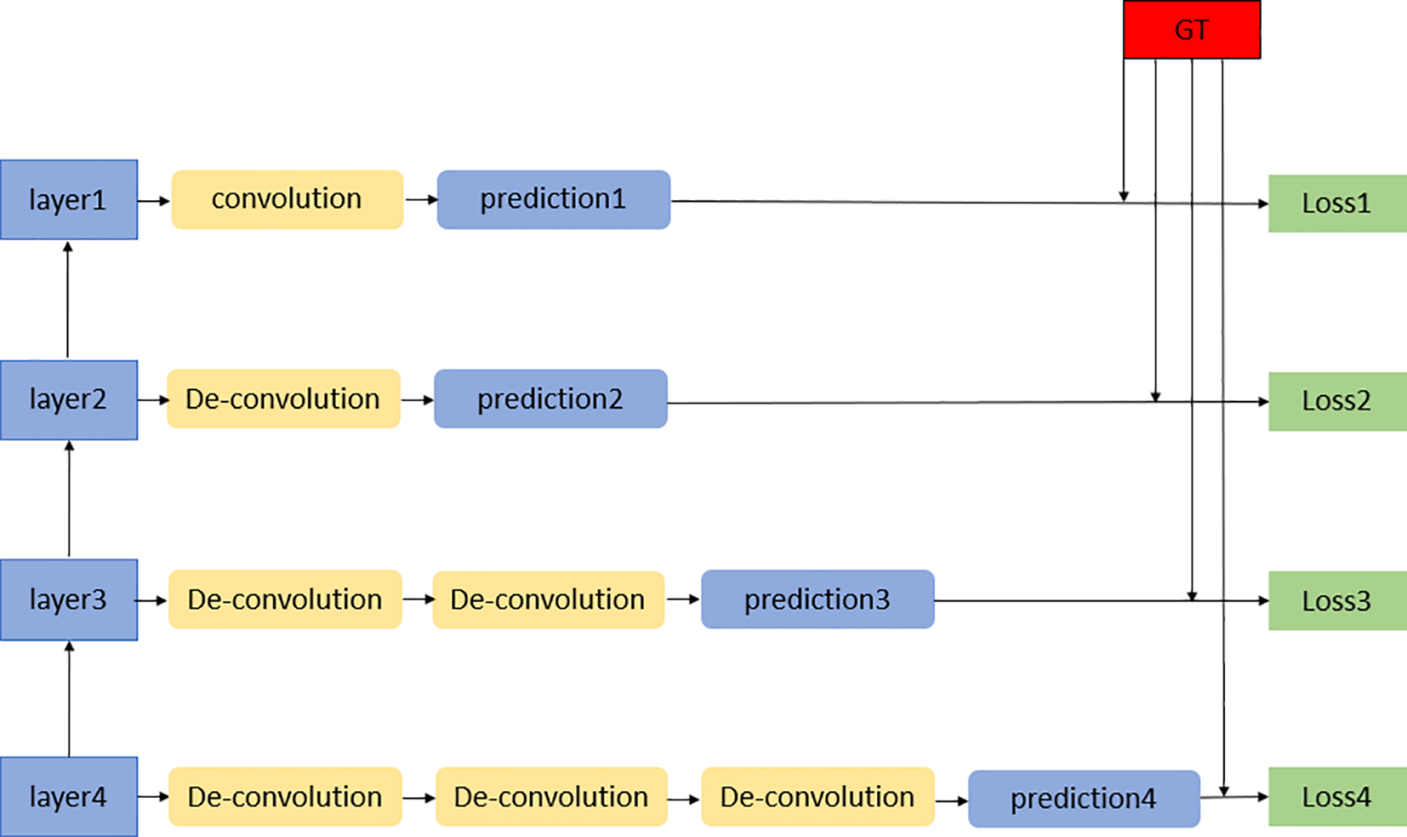
Deep supervision module.

Deep supervision is also called relay supervision. As shown in **Figure 1**, auxiliary loss functions are added to some intermediate hidden layers of the deep neural network as network branches to supervise the trunk network. Moreover, additional loss functions are added to the middle part of the network, and loss functions at different positions are summed by coefficients. The purpose is to train the features more fully and solve the problems of gradient disappearance and slow convergence of the deep neural network. As a training strategy, deep supervision was proposed in 2014 through deep supervision nets (DSNs) (14), which can improve the directness and transparency of the hidden layer learning process. To reduce the adverse effects of unstable gradient changes, we propose using display supervision to train the hidden layers in 3D DA-VNet. Specifically, we first use additional deconvolution steps to amplify some low-level and middle-level features. We then use softmax functions on these full-size characteristic volumes and obtain improved predictions. For these branch prediction results, we calculate their errors from the manual segmentation results. These auxiliary losses are integrated with the losses of the last layer to stimulate the backpropagation of gradients for more efficient parameter updates with each iteration.

We connect the volume features directly through the path to the last output layer as the primary network. Let $W^{l}$ be the weight of layer L of the primary network. Using W=( $W^{1},\;W^{2}$ , …, $W^{l}$ ), priority is given to the network weight set, and P( $t_{i}$ | $x_{i}$ ;w) represents the probability prediction of voxel $x_{i}$ after the softmax function in the last output layer. The negative log-likelihood loss function is expressed as:


$$p\left(t_{i}|x_{i};w\right)=\frac{{\text{e}}^{wx_{i}}}{\Sigma_{i}{\text{e}}^{wx_{i}}}$$

$$L\left(x;w\right)=-\sum_{x_{i}\in x}\log p\left(t_{i}|x_{i};w\right).$$

where x represents the training data, $t_{i}\;$ represents the target label of $x_{i}$ , and $x_{i}$ belongs to x . Additionally, we create an auxiliary intensive prediction layer called the branch network. Deep supervision is introduced through the branch network. When deep supervision is introduced in the m-th hidden layer, $w_{m}\;$ is used to represent the weight of the first m layers in the main network, and the weight of intensive prediction can be connected to the characteristic volume of the m-th layer through $\hat{w_{m}}$ . Then, the auxiliary loss function of deep supervision can be expressed as follows:


$$L_{m}=\left(x;w_{m},\hat{w_{m}}\right)=-\sum_{x_{i}\in x}\log p\left(t_{i}|x_{i};w_{m},\hat{w_{m}}\right)$$

Finally, we learn the weight w  and all $\hat{w_{m}}$ using the backpropagation algorithm by minimizing the following objective function:


$$L=L\left(x;w\right)+\sum_{m\in M}\eta_{m}L_{m}\left(x;w_{m},\hat{w_{m}}\right)$$

where $\eta_{m}$ represents the balance weight of $L_{m}$ , which decays during learning. M is the division of all the hidden layers with a deep supervision function. The first term corresponds to the prediction of the output in the last output layer, and the second term represents deep supervision. In each training iteration, the network’s input is the large-capacity data, and simultaneously, the error is backpropagated from these different weight losses.

The effectiveness of deep supervision can be proven through the following considerations. First, Qi et al. (15), in a liver segmentation challenge and a heart and large vessel segmentation challenge, used a deep supervision network to achieve a higher speed than the most advanced methods of competitive segmentation, thus proving the effectiveness of the deep supervision network. Zeng et al. (16) proposed a 3D UNet fully convolutional network with deep supervision for the segmentation of the proximal femur in 3D magnetic resonance (MR) images, and the experimental results proved the effectiveness of deep supervision. Zhu et al. (17), Yang et al. (18) and Bo et al. (19) applied deep supervision modules in different network structures to automatically segment the prostate gland. The final results proved that the deep supervision module could effectively address the optimization problem of gradient disappearance or explosion when training the 3D model, accelerate the convergence speed and improve the recognition ability.

### 2.5 Attention gate module

**Figure 2:**
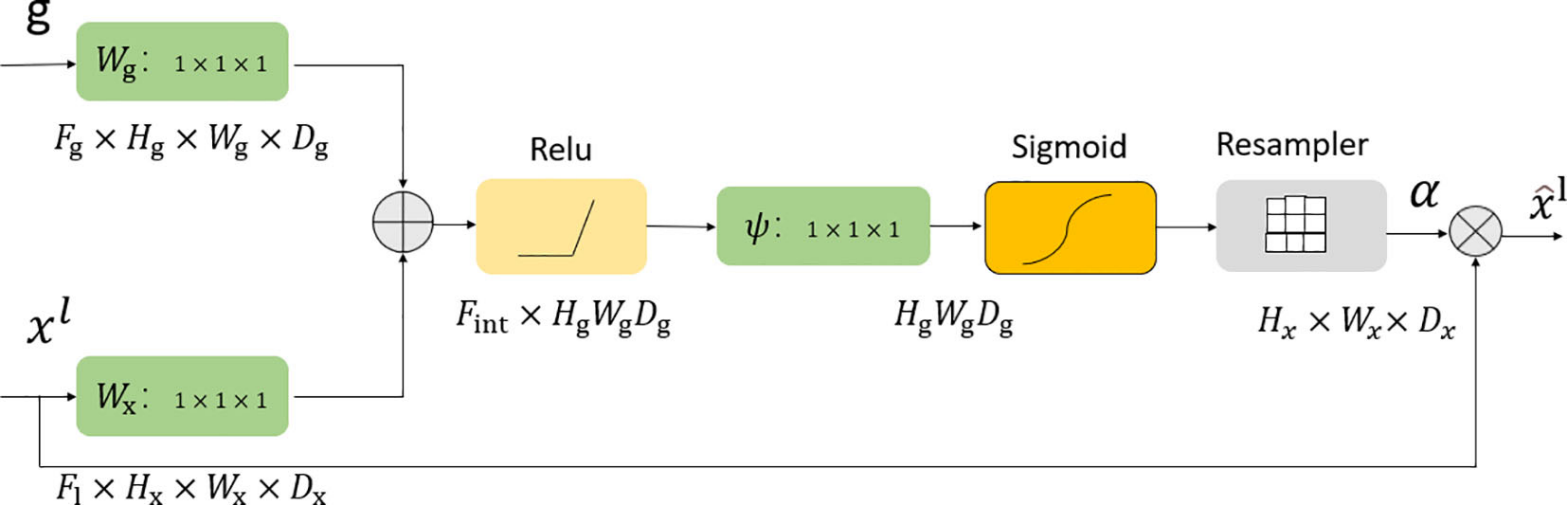
Attention gate module.

The attention gate (20) is shown in **Figure 2**, where g represents the feature map of the upward sampling and $x^{l}$ represents the feature map of the downwards sampling of the same layer. The two feature maps are adjusted to the same size and added together. The attention coefficient α can be obtained through ReLU, 1×1×1 convolution, a sigmoid function and resampling, and then through multiplication by $x^{l}$ , ${\hat{x}}^{l}$ can be obtained. The attention gate module can better ensure that attention is given to the prominent area and suppress the irrelevant background area, and it can be effectively embedded in the VNet network.

### 2.6 Network framework

**Figure 3:**
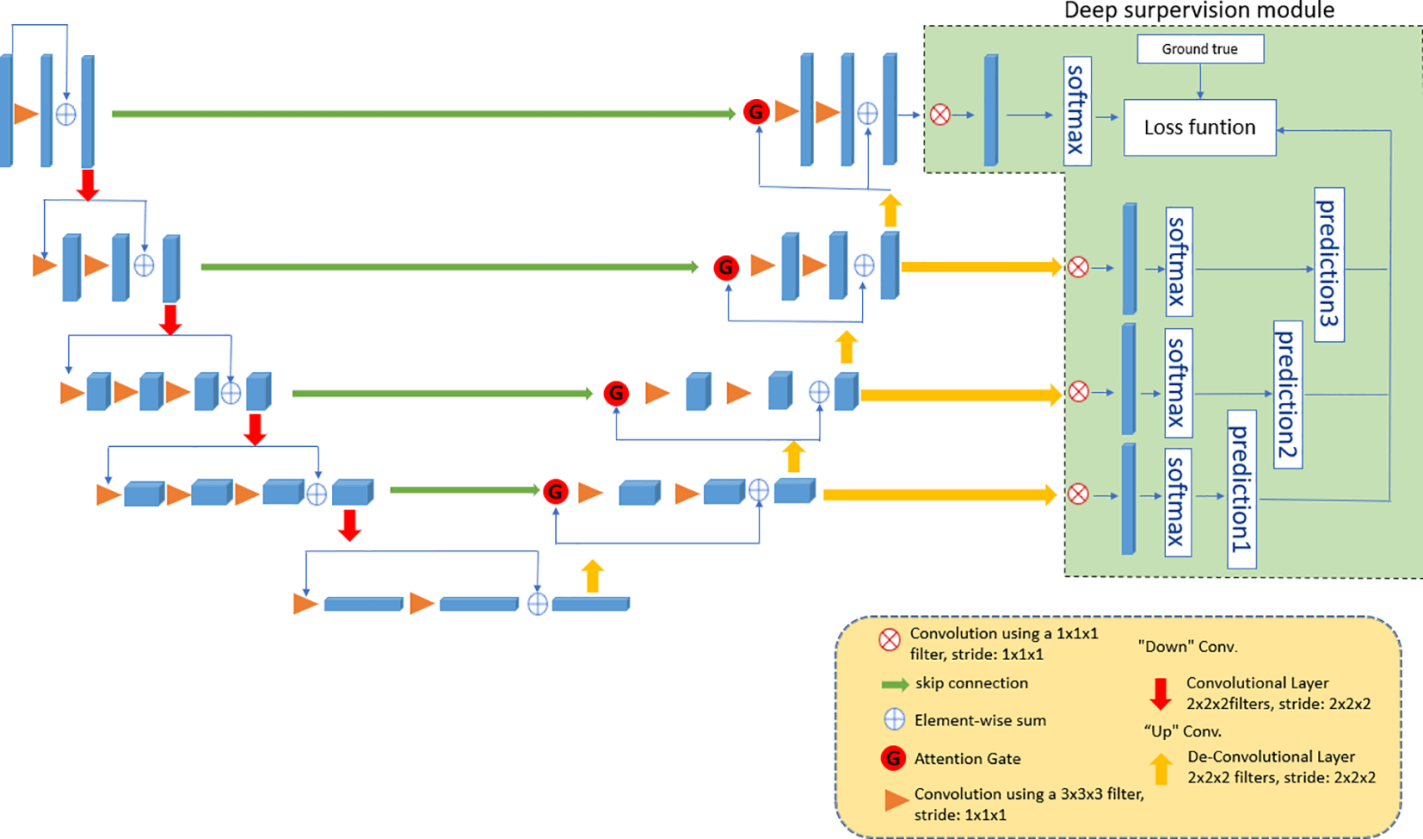
The framework of 3D DA-VNet.

VNet (21) usually consists of an input layer, a convolution layer, a lower sampling layer, an upper sampling layer and an output layer. The convolutional layer in the structure is used to learn image features, and local connections and weight sharing are used to reduce the number of parameters and the computational complexity. With the deepening of the network, layer-by-layer convolution can extract more abstract image features. As one of the key steps of pattern recognition, the quality of feature extraction directly affects the accuracy of image recognition. Through the study of VNet, we propose the 3D DA-VNet structure, which is more suitable for the 3D segmentation of MRI data of uterine fibroids. The overall framework of 3D DA-VNet is shown in **Figure 3**. In the upsampling stage, we combine the upsampling features with the downsampling features to introduce an attention gate. The attention gate module can better ensure that attention is given to the prominent area and suppress the irrelevant background area, and it can be effectively embedded in the VNet network. At the same time, in the upsampling stage, a deep supervision module is introduced to continuously upsample the output of each layer until the outputs are the same size as the training image. Then, a softmax function can be chosen to generate the contour probability maps followed by labelling each voxel. Accordingly, the losses of these intermediate layers together with that of the final output layer are combined for gradient backpropagation, which is used to identify more effective parameters with each iteration.

The parameter details of 3D DA-VNet are provided in **Table 2**. In the input layer, we input an image with a size of 128 × 128 × 48 and then convolve it with a 3 x 3 x 3 convolution kernel and activate it with PReLU. On this basis, standardization and a dropout rate of 0.01 are applied. The subsequent convolution operation is the same. Then, through downsampling, the stride of the convolution kernel is set to (1,2,2,2,1), and the deep features of the images are extracted. After continuous convolution and downsampling, the fifth layer is obtained, and its size is 8 × 8 × 3. After the deconvolution operation, the stride of the deconvolution kernel is (1,2,2,2,1), and the node is processed using the PReLU activation function. Therefore, the structure of the processing model retains the compatibility of the original network and lays the foundation for rapid iterative optimization. In the upsampling stage, the concatenation calculation is performed after each deconvolution operation. With this operation, the last layer uses softmax to predict values 0 and 1 for each pixel. Finally, the size of the image is recovered at 128 × 128 × 48.

**Table 2:** Parameter details of the proposed 3D DA-VNet.

Layer	Input Size	Components
Left-S 1	128*128*48	3*3*3,16, stride (1, 2, 2, 2, 1)
Left-S 2	64*64*24	3*3*3,32, stride (1, 2, 2, 2, 1)
Left-S 3	32*32*12	3*3*3,64, stride (1, 2, 2, 2, 1)
Left-S 4	16*16*6	3*3*3,128, stride (1, 2, 2, 2, 1)
Left-S 5	8*8*3	3*3*3,256, stride (1, 2, 2, 2, 1)
Right-S 4	16*16*6	3*3*3,128, stride (1, 2, 2, 2, 1)
Right-S 3	32*32*12	3*3*3,64, stride (1, 2, 2, 2, 1)
Right-S 2	64*64*24	3*3*3,32, stride (1, 2, 2, 2, 1)
Right-S 1	128*128*48	3*3*3,16, stride (1, 2, 2, 2, 1)
output	128*128*48	3*3*3,16, stride (1, 1, 1, 1, 1)

### 2.7 Evaluation metrics

We divided the data of 245 cases of uterine fibroids into a training set, testing set, and validation set at a ratio of 6:2:2, among which 147 cases were used for training, 49 cases were used for testing, and 49 cases were used for validation. Training and testing were carried out at the same time. After the model training was completed, it was verified by the validation set. The output of 3D DA-Vet was the prediction result of uterine fibroids. We used a single case as the input data to evaluate the performance of the model.

Before high-intensity focused ultrasound treatment of uterine fibroids, it is necessary to accurately locate uterine fibroids, so the accuracy of segmentation directly influences the therapeutic effect. However, for a pelvic image, the fibroid region only accounts for a very small part of the image, which often causes the segmented part to be neglected by the network, the output of the network being biased towards the background, and learning falling into a local extremum; ultimately, accurate results cannot be obtained. To avoid this problem, Spatial overlap was quantified using the Dice Similarity Coefficient (DSC) (22, 23). However, it’s important to note that DSC lacks a clear definition in cases where both compared volumes contain zero positive voxels, resulting in division by zero. The DSC calculation is as follows:


$$precision=\frac{TP}{TP+FP}$$

$$recall=\frac{TP}{TP+FN}$$

$$DSC=2*\frac{precision*recall}{precision+recall}$$

The IOU score is a standard performance measure for segmentation problems. Given an image, the IOU score gives the similarity between the predicted regions and ground-truth regions presented in the image, and it is defined by the following equation:


$$IOU=\frac{TP}{FP+TP+FN}$$

Sensitivity (24) measures the positive part of the voxel in the real background, which measures the ability to segment the fibroid region of the uterus:


$$Sensitivity=\frac{TP}{TP+FN}$$

Precision (25), also known as positive predictive value, refers to the accuracy of the segmentation of uterine fibroids:


$$Pr\text{e}cision=\frac{TP}{TP+FN}$$

where TP, FP and FN are the probabilities of true positives, false positives and false negatives, respectively. These evaluation indicators are all based on area and are sensitive to the divided internal filling area, while the Hausdorff distance (HD) (26) is based on distance and is sensitive to the divided boundary. The Hausdorff distance is a measurement used to describe the degree of similarity between two sets of points. Here, we use it to evaluate the relationship between the segmentation results and the real fibroid boundary distance.


$$HD=\max\left\{\max\underset{a\in gt\;b\in seg}{\min}\text{d}\left(a,b\right),\max\underset{a\in gt\;b\in seg}{\min}\text{d}\left(b,a\right)\right\}$$

where gt represents the ground truth, seg represents the segmentation result, A and B represent the voxels of the ground truth and the segmentation result, respectively, and D(a, b) represents the Euclidean distance between A and B.

## 3 Results and discussion

### 3.1 Effect of different convolution kernels and model structure

The convolution kernel is one of the most important concepts in deep learning. It has the advantage of weight sharing and translational invariance, and Yao Jin et al. (27) proposed that its size affects the number of model parameters and the information of extracted images. Therefore, we designed 3 × 3 × 3 and 5 × 5 × 5 convolution kernels. In addition, minor adjustments to the overall structure will affect the final result, including the number of convolutions for each layer. We evaluated our proposed 3D DA-VNet on datasets using DSC, IOU, sensitivity, precision, and HD, and present the results of the 6 experiments in **Table 3**.

**Table 3:** Quantitative evaluation results of the dataset.

the Model Structure	Convolution Kernel Size	DSC	IOU	sensitivity	precision	HD(mm)
(1,2,3,3,3,3,3,2,1)	3*3*3	0.876 ± 0.038	0.781 ± 0.059	0.867 ± 0.070	**0.893** ± **0.070**	11.34 ± 7.216
	5*5*5	0.858 ± 0.040	0.754 ± 0.060	0.858 ± 0.074	0.870 ± 0.082	12.01 ± 6.842
(1,2,2,2,2,2,2,2,1)	3*3*3	0.870 ± 0.039	0.772 ± 0.060	0.863 ± 0.076	0.888 ± 0.075	12.07 ± 7.650
	5*5*5	0.862 ± 0.053	0.773 ± 0.066	0.875 ± 0.077	0.875 ± 0.075	11.45 ± 7.163
(1,2,3,3,2,2,2,2,2)	3*3*3	**0.878** ± **0.037**	**0.784** ± **0.057**	**0.879** ± **0.072**	0.885 ± 0.067	**11.18** ± **7.181**
	5*5*5	0.857 ± 0.037	0.752 ± 0.057	0.863 ± 0.085	0.865 ± 0.080	12.36 ± 7.840

Bold numbers indicate the best results.

### 3.2 The influence of different network training on the results

**Figure 4:**
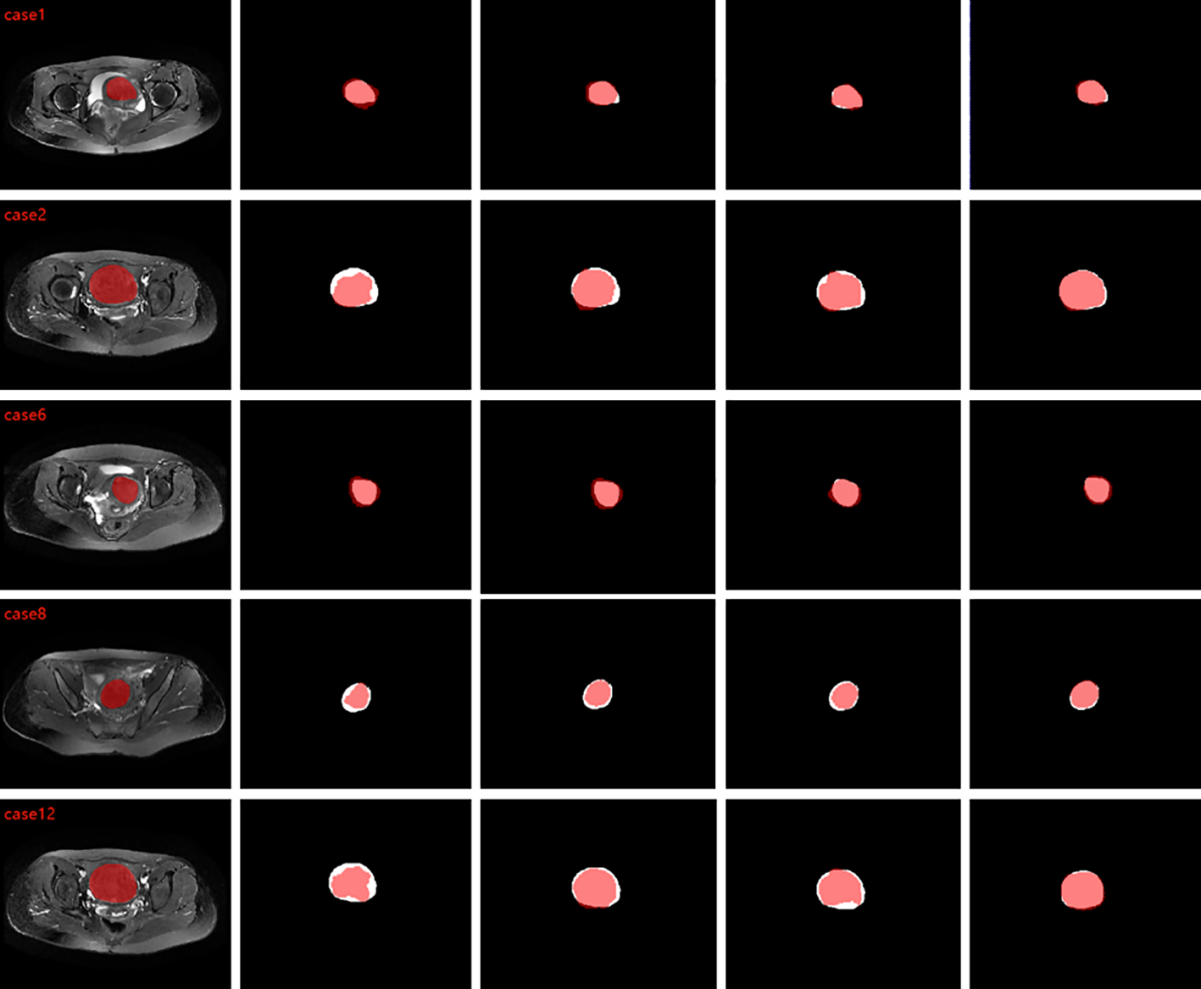
From left to right are the comparison graphs of the segmentation results of ground truth and 3D VNet, 3D DS-VNet, 3D attention VNet, and 3D DA-VNet, where the white area is ground truth and the red area is the segmentation result.

To validate the proposed method, we tested T2WI MRI of 49 patients and segmented different shapes of uterine fibroids. To compare the segmentation results with the ground truth, we calculated DSC, IOU, sensitivity, accuracy, and HD, and **Table 4** shows the results of comparing the algorithm proposed in this paper with 2D UNet, 2D VNet, 3D UNet, 3D VNet, 3D deep supervision VNet (3D DS-VNet) and 3D attention VNet. The DSC of our proposed method reached 0.878. The IOU was 0.784, the sensitivity was 0.879, the precision was 0.885, and the HD was 11.18 mm. Due to the large amount of 3D MRI segmentation data, the average segmentation time of a uterine fibroid was 7 seconds. **Figure 4** compares the segments that result in the segmentation. As seen from **Figure 4**, compared to 3D DS-VNet and 3D attention VNet, 3D DA-VNet has a better segmentation effect at the fibroids’ boundary and has a more accurate segmentation of uterine fibroids, which gives it advantages in the image processing of uterine fibroids. For fibroids with irregular shapes and small sizes, the segmentation effect is better.

**Table 4:** Testing results of uterine fibroids segmentation on different networks.

Method	DSC	IOU	Sensitivity	Precision	HD(mm)
2D Unet	0.725 ± 0.162	0.614 ± 0.133	0.724 ± 0.141	0.789 ± 0.153	28.38 ± 12.760
3D Unet	0.740 ± 0.089	0.626 ± 0.115	0.739 ± 0.080	0.805 ± 0.143	27.90 ± 10.496
2D Vnet	0.783 ± 0.140	0.697 ± 0.129	0.787 ± 0.148	0.857 ± 0.163	20.85 ± 11.014
3D Vnet	0.833 ± 0.062	0.740 ± 0.074	0.848 ± 0.084	0.866 ± 0.106	13.70 ± 7.164
3D DS-Vnet	0.846 ± 0.051	0.750 ± 0.070	0.863 ± 0.076	0.862 ± 0.096	12.55 ± 7.218
3D attention Vnet	0.862 ± 0.043	0.772 ± 0.058	0.873 ± 0.073	0.878 ± 0.075	12.23 ± 7.767
3D DA-Vnet	**0.878** ± **0.037**	**0.784** ± **0.057**	**0.879** ± **0.072**	**0.885** ± **0.067**	**11.18** ± **7.181**

The best results (expressed as mean ± standard deviation) are shown in bold.

### 3.3 Discussion

We compared the size and number of different convolution kernels, as shown in **Table 3**. From the final results, we chose the size of the convolution kernel as 3x3x3, with (1, 2, 3, 3, 2, 2, 2, 2, 2) as the structure. We also compared different 2D and 3D networks, as well as UNet and VNet networks. The final results in **Table 4** show that the segmentation effect of our proposed 3D DA-VNet is the best, and they also prove that the deep supervision module and the attention gate module are effective. Additionally, **Figure 4** confirms that our predicted target regions have a good overlap with the standard manual segmentation results given. To better understand the results of our segmentation, we compared the results of the segmentation of uterine fibroid MRI data with those of other studies. The corresponding comparisons are shown in **Table 5**. These are traditional segmentation methods, including the fuzzy C-means algorithm, split-and-merge, level set segmentation, etc. Their segmentation results are also good, but these traditional methods require complex preprocessing and postprocessing of the data. Segmentation is not performed synchronously, which makes the segmentation process inefficient. In conclusion, although these conventional methods have some merits in terms of performance, they show some practical limits in a clinical setting. **Table 6** gives a comparison with some deep learning segmentation methods. The existing deep learning methods for segmenting uterine fibroids all segment 2D slice data, which loses considerable image spatial information and is not conducive to the effective segmentation of 3D images, so the segmentation accuracy is not high. To obtain more in-depth information, to better meet the needs of clinical diagnosis, and to propose a more effective 3D image processing method, we propose the automatic segmentation of uterine fibroids using 3D DA-VNet based on deep supervision and attention gates. The deep supervision module allows features to be more fully trained by deep feature supervision, thus improving the segmentation performance. The attention gate module improves segmentation performance by ensuring that more attention is given to the region of interest while suppressing irrelevant background regions, and the final DSC value in the testing set is 0.878.

**Table 5:** Comparison of traditional methods for segmenting uterine fibroids.

Authors	Year	Dataset size	Dataset source	Algorithm	Evaluation metrics
N. Ben-Zadok et al (28)	2009	4	private	Level set segmentation, User feedback	TPE=0.999
A. Fallahi et al (29)	2010	5	private	fuzzy C-means algorithm and morphological operations,modified possibilistic fuzzy c-mean	SI=0.799
A. Fallahi et al (30)	2011	10	private	Fuzzy C-Mean (FCM) method and morphological operations	SI=0.800
H. Khotanlou et al (31)	2014	15	private	Chan–Vese level set method geometric shape prior model	SI=0.877
Xu M et al (32)	2015	42	private	split-and-merge	SI=0.876
Militello C et al (33)	2015	15	private	Unsupervised Fuzzy C-Means clustering and iterative optimal threshold selection algorithms	SI=0.887
Rundo L et al (34)	2016	14	private	Dataset normalization Region splitting and merging Seed-region refinements	SI=0.876
Rundo L et al (35)	2019	18	private	Iterative Optimal Threshold Selection (IOTS) Split-and-Merge(SM),Region Growing(RG)	DSC=0.873 DSC=0.875
Ning G et al (36)	2020	320	private	CNN, encoder and decoder paths	DSC=0.812

**Table 6:** Comparison of methods for deep learning segmentation of uterine fibroids.

Authors	Year	Dataset size	Dataset source	Network	DSC	IOU	Precision	Recall (Sensitivity)
Ours	2022	249	private	3D DA-Vnet	0.878	0.784	0.885	0.879
Kurata Y et al (7)	2019	122	private	U-net	0.820	—	—	—
Tang et al (8)	2020	93	private	AR-Unet	0.904	0.844	—	0.886
Zhang et al (9)	2020	297	private	HIFUNet	0.835	—	0.845	0.837
Wang et al (37)	2024	550	private	nn-Unet	0.956	—	—	—

However, we can see from **Table 6** that compared with AR-Unet and nn-Unet results, our results are not outstanding. The main reason for our analysis lies in the difference of images. We used transverse magnetic resonance images, while Tang (8) and Wang (37) used sagittal magnetic resonance images. In anatomy, sagittal images can more clearly show the long axis of the uterus and the uterine lumen-fibroid interface, while the transverse section is easy to truncate the fibroid shape, and the boundary of the transverse expanded fibroids is blurred, making segmentation difficult. Our study mainly focuses on the efficacy of HIFU ablation in the treatment of uterine fibroids. We will use different MRI sequences, which will all have transverse images, but not necessarily sagittal images. Therefore, we choose to develop a model for segmenting transverse images, so as to better combine clinical treatment in our subsequent studies. On the other hand, the lack of data is also a factor that makes the results low.

Judging from the results, the algorithm we proposed demonstrates a certain efficacy in the segmentation of uterine fibroids. However, our study is not without limitations that may impact the generalizability and accuracy of the findings. Firstly, the sample size of our data is relatively small; specifically, 245 MRI images may be insufficient for training deep learning models given the complexity inherent in MRI data. The ability to generalize this model to larger and more diverse datasets remains uncertain. Secondly, our magnetic resonance imaging (MRI) dataset is limited to a single series; this study relies solely on T2-weighted imaging. Uterine fibroids may exhibit different characteristics across other MRI sequences or imaging modalities, which constrains the clinical applicability of this model when applied under varying imaging conditions. Finally, this study does not compare with current state-of-the-art segmentation techniques and may underestimate the performance advantages or limitations of these techniques. While the methods employed in this study perform well under certain conditions, more room for improvement and potential challenges may be found compared to the latest techniques. Therefore, future research will incorporate these into the research scope to ensure the advanced nature and optimization potential of the method. Additionally, due to the presence of significant surrounding tissue around uterine fibroids, caution must be exercised when utilizing high-intensity focused ultrasound for ablation procedures so as not to damage adjacent structures. Therefore, multi-organ segmentation encompassing uterine fibroids along with related anatomical structures such as the uterus and spine represents a more aligned approach with actual clinical needs—this also constitutes a promising direction for future research endeavors.

## 4 Conclusions

This study uses deep learning to achieve automatic segmentation of uterine fibroids magnetic resonance data. Most of the previously proposed deep learning models first convert 3D MRI data into 2D image slices and then use them to train and optimize the network, which wastes considerable time and does not accord with the 3D characteristics of the images themselves. For the 3D DA-VNet proposed in this paper, the input layer is a 3D matrix, which can speed up training progress and improve training efficiency. In addition, this paper introduces a deep supervision module and attention gate. The use of the deep supervision module allows the layers to be more fully trained and addresses the depth gradient disappearance and the slow convergence speed of the neural network, and integration of the attention gate can better ensure focus on the area of interest and suppression of irrelevant background regions. The experimental results for the same data set and platform show that the accuracy of the proposed method is significantly improved compared to other segmentation methods. It is believed that with improvement in segmentation accuracy, automatic segmentation of uterine fibroids can help doctors make more accurate judgments, improve work efficiency, and reduce the rate of misdiagnosis.

## Data Availability

The raw data supporting the conclusions of this article will be made available by the authors, without undue reservation.
